# A tale of two epidemics: gender differences in socio-demographic characteristics and sexual behaviors among HIV positive individuals in Mexico City

**DOI:** 10.1186/s12939-015-0286-4

**Published:** 2015-12-16

**Authors:** Sergio Bautista-Arredondo, Edson Servan-Mori, Fenella Beynon, Andrea González, Patricia Volkow

**Affiliations:** National Institute of Public Health, Cuernavaca, Mexico; Mexico City’s AIDS Program, Mexico City, Mexico; National Institute of Oncology, Mexico City, Mexico

**Keywords:** Gender inequality, Social vulnerability, Risk behavior, HIV, Mexico City

## Abstract

**Background:**

To date, the HIV epidemic in Mexico has been concentrated mainly among men who have sex with men, butheterosexual transmission, particularly to women, is increasingly important. This study examine gender differences in socio-demographic characteristics and risk behaviors of HIV positive individuals in Mexico City.

**Methods:**

We analyzed data from a cross-sectional survey of 1,490 clinic patients (male:female ratio 8:1) with HIV inMexico City in 2010. We examined socio-demographic characteristics, risk behavior, and history of HIV infection.From multivariate non-linear probability (probit) models we calculated predicted probabilities by sex of several outcomes: marginalization, demographic and sexual risk behaviors.

**Results:**

Significant differences were found between men and women. Multivariate models suggest that women had lower schooling levels; were less likely to have been employed in the past month and earn more than the minimal wage; more likely to have children, to have been sexually abused, to never have used condoms and to report having been infected by a stable partner. Additionally, women were less likely to report having a partner with a history of migration to the USA and to have engaged in transactional sex.

**Conclusion:**

Significant differences exist between men and women with HIV in Mexico City in terms of their socioeconomicand behavioral profiles, which translate into differences in terms of exposure to HIV infection. Women face social and economic vulnerability while men tend to have riskier sexual behavior. Gender issues must be approached in prevention and treatment efforts, using diverse methods to target those most vulnerable and at risk.

## Background

In Mexico, like much of Central and South America, the HIV epidemic is defined as *concentrated* [1]. Nationally, prevalence is low (about 0.3 %), but ranges from 10 to 17 % among men who have sex with men (MSM) and from 5 to 8 % among intravenous drug users (IDUs) [1–3]. Higher than average prevalence is also seen among sex workers, transgender, transsexual and transvestite persons and prison inmates [4, 5]. Of the 220,000 people estimated to be infected with HIV in Mexico, almost two thirds are MSM [1, 2].

However, heterosexual transmission increasingly plays an important role in the epidemic in Mexico, accounting for about 50 % of new infections by 2010, compared with 10 % in the early years of the epidemic [3]. Similarly, male to female mortality ratios have shifted from 12:1 before 1988 to 4.9:1 in 2012 [6]. Both global and local explanations have been offered for this trend, which is not unique to Mexico. Among them, the Pan-American Health Organization (PAHO) concluded that women are more vulnerable to infection, both biologically and socially, while men tend to be at a greater risk due to “pro-active” behavior [7].

Women in Mexico are vulnerable to HIV infection mainly as a result of unprotected sex with stable partners, who are at risk through unprotected sex with multiple or high risk partners, particularly other men [8–10]. Migration also plays a role in the epidemic, primarily through young men migrating to the U.S. This type of migration is linked with adopting more risky behaviors, social isolation and seeking comfort in new sexual partners [11, 12], which has implications for partners at home – most of them women. When men return home, after unprotected sex with casual partners in the U.S., condom use with their partners is very low [13–15].

Intimate partner violence (IPV) is also an important factor in the spread of HIV to women in stable relationships, by reducing women’s ability to negotiate condom use and also by increased transmission risk from sexual IPV [16–19]. In Mexico, 47.1 % of women have experienced some form of IPV, of which 29.9 % was physical or sexual [20].

In Latin America, high HIV prevalence among MSM, combined with low risk perception and poor availability of information and access to preventive services due to stigma, lead to increased risk [21]. Despite some evidence of more consistent condom use among MSM [22–24], greater risk has been attributed to increased partner concurrency, longer periods of new partner acquisition and greater age variation in partnerships [25]. Bisexual and non-gay identifying MSM, more common in cultures with high levels of stigma about homosexuality, are at higher risk of HIV because of a complex array of factors: in particular, poverty, stigma, drug use and sex work [26]. Male-to-female transgender individuals are particularly stigmatized leading to an increase in risky behavior, including sex work, unprotected sex and needle sharing for the purpose of using illicitly obtained hormones [4, 27].

Despite extensive literature documenting patterns behind the HIV epidemic in Mexico, we have little evidence about gender differences in HIV risk behaviors. The purpose of this study is to describe gender differences in socio-demographic characteristics and risk behaviors of a large, representative population of HIV positive individuals receiving care and treatment in Mexico City. With improved understanding of the differing characteristics, risk factors, and behaviors between men and women in this population, both preventive and treatment services can be tailored to improve outcomes.

## Methods

### Data collection

This study examines cross-sectional data collected at the *Clinica Especializada Condesa* (CEC) between October 2010 and March 2011 by the National Institute of Public Health of Mexico (INSP). The project was reviewed and approved by INSP Committees of Research, Biosecurity and Ethics (code: 1452895478126086).

The CEC is among the largest outpatient clinics in Latin America specializing in HIV/AIDS. It provides medical care for HIV-positive uninsured Mexicans at no charge. At the end of 2011 it had more than 6,500 active patients and carried out about 5,600 consultations per month. According to CEC estimates, 40,000 people were living with HIV in the city in 2012 (17 % of the national total) and there were roughly 2,800 new infections in 2013 (30 % of all new infections in Mexico) [28].

During the data collection period, all patients in the CEC waiting rooms were invited to participate in this study. Trained INSP personnel explained the study orally and in writing. Oral informed consent was documented, and no record of names was kept of those who did or did not participate, to ensure confidentiality. Following informed consent, participants completed a questionnaire in Spanish using the Audio Computer Assisted Self Interview (ACASI) method. Participants used a touchscreen computer to view and answer questions while also listening to a pre-recorded audio file with the same information.

Individual-level data was collected on socio-demographic background, ethnicity, risk behavior and sexual practices, history of infection, sexual violence, social support [29], self-placement on the social ladder [30], knowledge of HIV, and alcohol and/or drug use at sexual encounters. During the implementation of the survey, study staff was on hand to answer any queries and to provide support to interviewees. Participants could decline to answer any question at any time.

### Sample

In order to have a consistent analytical sample through all the analysis, we excluded participants for whom data was incomplete in any variable of interest. The final analytical sample included 1,490 participants (1,326 men and 164 women), around 85 % of the total sample. 112 men and 155 women were excluded. We found differences between those individuals included in the analysis and those who were excluded. The final analytical sample was characterized by a higher percentage of men, a higher percentage of employed individuals, a lower percentage of people self-identified as gay or lesbian, a higher percentage of people self-identified as heterosexual, and a higher proportion of single people. These differences and their implications on the conclusions will be discussed in the discussion section of the manuscript.

### Measurement

#### Dependent variables

We analyzed the following socioeconomic characteristics as dependent variables: marital status (married/cohabiting vs. any other); number of children (having at least 1); whether they were living alone at the time of the interview; employment status during the past month; wage (more than the minimal wage vs. less); higher educational level (having completed high school level or higher); lower educational level (6 years or less); and an index of social support based on the Duke’s functional questionnaire of perceived social support – previous studies have shown the validity of this questionnaire for the Mexican population [29]. The respondents were classified into two groups: low social support (score between 0 and 31), and high social support (score between 32 and 55).

We also analyzed the following sexual behaviors and characteristics as dependent variables: sexual identity (heterosexual vs. gay/lesbian or bisexual); whether the presumed route of HIV infection was by a stable partner; age of first sexual intercourse; and number of sexual encounters in the previous month. Dependent variables related to sexual violence and risk behavior history before HIV diagnosis included: experience in transactional sex (ever); frequency of condom use at sexual encounters (never vs. sometimes, frequently, almost always, always), alcohol or drug use at sexual encounters (never vs. sometimes, frequently, almost always, always); and history of sexual abuse (at least once). Finally, prior migration to the U.S. and prior incarceration were also analyzed as dependent variables.

#### Control variables

All models were controlled for the following socioeconomic and risk characteristics: sex, age, indigenous status, living alone, marital status, number of children, educational level, employment status during the past month, prior incarceration, prior migration to the U.S. Sexual identity was included as a control variable in all the models with socioeconomic variables as dependent variables. When any of these control variables was a dependent variable in the model, it was nor included as control.

#### Other variables included in the descriptive analysis (differences of means)

In addition to the dependent and control variables described before, the following variables were not included in the regression analyses, but were included in the comparison of means between men and women presented in Tables 1, 2 and 3.

**Table 1:** Socioeconomic and demographic characteristics of study participants

*N* = 1,490	Women	Men	*p*-value*
	11.2 %	88.8 %	
	Mean or percentage [CI-95 %]		
Age	35.6 [34.0,37.1]	34.8 [34.3,35.3]	0.35
Highest education level			
Pre-primary or nothing	8.38 [4.96,13.7]	5.74 [4.61,7.14]	<0.001
Primary (1 - 6 yrs of schooling)	29.3 [22.9,36.7]	4.54 [3.53,5.80]	
Secondary (7 – 9 yrs of schooling)	32.3 [25.7,39.8]	16.4 [14.5,18.5]	
High school (10 – 12 yrs of schooling)	14.4 [9.80,20.6]	25.7 [23.4,28.1]	
Higher education	15.6 [10.8,21.9]	47.6 [44.9,50.3]	
Marital status			
Single	32.9 [26.2,40.4]	72.3 [69.8,74.6]	<0.001
Married/Cohabiting	41.9 [34.7,49.5]	23.7 [21.4,26.0]	
Divorced/Separated	7.78 [4.50,13.0]	3.55 [2.67,4.70]	
Widow or widower	17.4 [12.3,23.9]	0.53 [0.23,1.11]	
Partner died of AIDS (among widows or widowers)	85.2 [66.9,94.7]	85.7 [46.7,99.5]	0.97
Number of children			
0	18.0 [12.8,24.5]	87.6 [85.7,89.3]	<0.001
1	26.9 [20.8,34.2]	4.84 [3.80,6.14]	
2	23.4 [17.6,30.4]	4.31 [3.33,5.55]	
3 or more	31.7 [25.1,39.2]	3.25 [2.41,4.36]	
Indigenous	11.4 [7.38,17.3]	7.48 [6.17,9.04]	0.08
Living alone	5.39 [2.72,10.1]	19.0 [16.9,21.2]	<0.001
Worked the past month	67.1 [59.6,73.8]	75.7 [73.4,78.0]	0.02
Type of contract			
Temporary	14.0 [8.72,21.7]	21.6 [19.1,24.3]	<0.001
Permanent	7.02 [3.41,13.4]	17.4 [15.2,19.9]	
None	78.9 [70.5,85.5]	61.0 [57.9,64.0]	
Wage [mean, sd] in US$	172.1 [150.6,193.5]	391.8 [366.3,417.2]	<0.001
Duke-UNK’s Social Support classification			
High social support	62.1 [53.6,69.9]	72.0 [69.2,74.6]	0.03
Self-placement on the social ladder			
I to III	15.3 [8.01,26.7]	5.07 [3.30,7.67]	<0.001
IV to VI	39.0 [27.6,51.8]	21.3 [17.6,25.5]	
VII to IX	45.8 [33.7,58.3]	73.7 [69.2,77.7]	
Previously in prison	2.53 [0.77,6.55]	4.68 [3.64,5.98]	0.22
Previously migrated to the USA	2.55 [0.77,6.59]	11.8 [10.2,13.7]	<0.001
Sexual identity			
Gay/Lesbian	1.20 [0.05,4.54]	80.3 [78.0,82.3]	<0.001
Heterosexual	91.0 [85.6,94.6]	11.0 [9.45,12.8]	
Bisexual	7.78 [4.50,13.0]	8.69 [7.29,10.3]	

Footnote: *Tests for differences in means or proportions. We used bivariate *t*-test for continuous variables and Chi-square test for categorical variables to compare men and women

**Table 2:** History of HIV infection among study participants

	Women	Men	*p*-value*
	(*N* = 167)	(*N* = 1,323)	
	Percentage [CI-95 %]		
Presumed route of HIV infection			
Blood transfusion	6.98 [3.55,12.9]	1.53 [0.95,2.42]	<0.001
Sharing syringes	1.55 [0.07,5.83]	0.51 [0.21,1.14]	
Sex without condom	84.5 [77.2,89.8]	82.5 [80.2,84.6]	
Condom failure	3.10 [0.95,7.97]	12.1 [10.3,14.1]	
I was forced to have sex	3.10 [0.95,7.97]	3.06 [2.21,4.22]	
I forced someone to have sex	0.78 [0.00,4.69]	0.34 [0.10,0.90]	
Infected by a stable partner	69.6 [61.0,77.0]	44.0 [41.0,46.9]	<0.001
Migrant/military/prison background of the person who infected you			
Military or police	18.7 [12.7,26.6]	3.77 [2.64,5.35]	<0.001
Prison experience	16.8 [11.0,24.9]	3.42 [2.29,5.07]	<0.001
Migrant to the USA	17.3 [11.3,25.5]	27.4 [24.1,31.1]	0.01
Migrant, military or prison background	41.5 [33.1,50.3]	24.4 [21.6,27.4]	0.01
Reason for getting HIV test			
Unprotected sex	12.6 [8.13,18.9]	19.9 [17.7,22.2]	<0.001
Tested while hospitalized	14.6 [9.76,21.1]	13.9 [12.0,15.9]	
Medical advice	12.6 [8.13,18.9]	18.2 [16.1,20.4]	
Requested by employer	0.66 [0.00,4.03]	1.62 [1.04,2.51]	
When donating blood	3.97 [1.65,8.59]	3.57 [2.66,4.77]	
HIV positive partner	25.8 [19.5,33.4]	15.5 [13.6,17.6]	
Shared injecting equipment	0.66 [0.00,4.03]	0.16 [0.00,0.63]	
Routine test (frequent tester)	1.32 [0.06,5.01]	7.54 [6.19,9.16]	
Other	19.9 [14.2,27.0]	19.7 [17.6,22.0]	
In pregnancy	7.95 [4.48,13.5]	----	

Footnote: *Tests for differences in means or proportions. We used bivariate *t*-test for continuous variables and Chi-square test for categorical variables to compare men and women

**Table 3:** Sexual violence and risk behaviour history before HIV diagnosis among study participants

	Women	Men	*p*-value*
	(*N* = 167)	(*N* = 1,323)	
	Mean or percentage [CI-95 %]		
Any time in life			
Frequently, almost always or always	3.85 [1.19,9.79]	19.4 [16.5,22.6]	<0.001
Sex with men only	91.7 [85.3,95.6]	80.6 [78.1,82.9]	0.01
Sex with women only	4.13 [1.53,9.56]	9.46 [7.82,11.4]	
Sex with men and women	4.13 [1.53,9.56]	9.93 [8.26,11.9]	
Risk behavior at sexual encounters before diagnosis			
Frequency of condom use			
Never	73.8 [65.3,80.8]	49.8 [46.7,52.8]	<0.001
Sometimes	11.5 [6.84,18.5]	23.1 [20.6,25.8]	
Frequently, almost always or always	14.8 [9.45,22.2]	27.1 [24.5,29.9]	
Frequency of alcohol use			
Never	70.7 [62.1,78.1]	55.2 [52.1,58.2]	<0.001
Sometimes	18.7 [12.7,26.6]	27.9 [25.3,30.8]	
Frequently, almost always or always	10.6 [6.16,17.4]	16.9 [14.7,19.3]	
Frequency of drug use			
Never	86.7 [79.3,91.7]	79.8 [77.2,82.1]	0.20
Sometimes	7.50 [3.82,13.8]	10.8 [9.07,12.9]	
Frequently, almost always or always	5.83 [2.65,11.8]	9.38 [7.73,11.3]	
Frequency of both drug and alcohol use			
Never	88.5 [81.5,93.2]	80.2 [77.7,82.6]	0.06
Sometimes	4.92 [2.05,10.5]	11.4 [9.54,13.4]	
Frequently, almost always or always	6.56 [3.18,12.6]	8.41 [6.86,10.3]	
Sexual abuse			
Sexually abused at least once	17.9 [12.7,24.6]	12.6 [10.9,14.6]	0.09
Average (sd) number of times sexually abused	11.8 [4.66,18.9]	3.69 [2.72,4.67]	0.02
Age when first sexually abused	17.9 [13.6,22.2]	13.5 [11.9,15.2]	0.05
Number of sexual encounters in the last month ^a^	9.30 [6.57,12.0]	9.18 [8.30,10.1]	0.94
Age of first sexual intercourse	16.9 [16.3,17.6]	15.5 [15.2,15.7]	<0.001

Footnote: *Tests for differences in means or proportions. We used bivariate *t*-test for continuous variables and Chi-square test for categorical variables to compare men and women

^a^78 % of women reported having frequent sex with regular partners (42.9 % among males), the rest reported frequent sex with an occasional or one-time partner (data not shown)

We asked the participants their relative position on the social ladder as they perceive it. The social ladder depicts nine positions and respondents classify themselves with respect to their social environment. We classified their responses in three categories: positions I to III as low, positions IV to VI as middle, and positions VII to IX as high [30]. With respect to history of HIV infection, we analyzed information on the presumed route of HIV infection (blood transfusion, sharing syringes, sex without condom, condom failure), other characteristics of the person who presumably infected the patient (military/police, prisoner, migrant to the USA), and the reason for getting an HIV test (unprotected sex, tested while hospitalized, medical advice, requested by employer). Finally, an additional measure of sexual behavior included was prior sexual experience with men/women only vs. with both men and women.

### Analytical strategy

Descriptive socio-economic and demographic backgrounds as well as the history of HIV infection, and of sexual violence and risk behavior before HIV diagnosis by sex are presented and tested for statistical differences. For categorical variables, chi-square tests were performed to determine statistical independence between men and women in at least one category. Similarly, we used t-tests to determine independence in continuous variables.

Tests for gender differences in a multivariate context were performed modeling the variable sex (male/female) as the primary predictor of marginalization and risk indicators using multivariate, non-linear probability (probit) models. From this models we estimated adjusted probabilities and confidence intervals [95 % CI] for two types of outcomes variables:

- Eight marginalization indicators: marital status (cohabiting or married), having at least one child, living alone, employment status during the previous month, income level (greater than or equal to the minimum wage in Mexico City [31]), level of education (high school or higher and 6 years or less), and perception of high social support.
- Ten variables related to risk behavior indicators include: sexual identity (heterosexual vs. other), infected by stable partner, age of first sexual intercourse, number of sexual encounters in the previous month, ever engaged in transactional sex, used condom before HIV diagnosis (never vs. at least sometimes), alcohol use before sexual encounters (never vs. at least sometimes), sexual abuse at least once, previous migration to the U.S., and prior incarceration.

The analytical strategy consists on predicting adjusted probabilities of each outcome of interest by sex, while holding all covariates at their mean levels. This strategy was implemented using the *margins* command in Stata. All analyses were performed using Stata SE 13.1 software [32].

## Results

Of the 1,490 participants, 1,326 (89 %) were men and 164 (11 %) women. The average age of 35 years (Table 1), and the male:female ratio was 8:1, which is consistent with the total population of the CEC (7.7:1) at the time of the survey.

In bivariate analyses (Table 1), we found several statistically significant differences (*p* < 0.05) between men and women. Seventy-three percent of men had 10 or more years of schooling compared with 30 % of women; 72 % of men were single, compared with 33 % of women. While 88 % of men were childless, only 18 % of women reported not having children. Speaking an indigenous language (a commonly used proxy for extreme social vulnerability in Mexico) was more common among women than men. Living alone was more common among men than women.

Further differences were found in terms of labor and social placement outcomes. First, 75 % of men indicated having worked during the past month, and among them an average monthly salary of US$392 was reported. In comparison, 67 % women worked during the past month, with an average monthly salary of US$172. On the other hand, 72 % of men perceived having high social support vs. 62 % of women, and 74 % of men placed themselves between tiers VII and IX of the social ladder vs. 46 % of women.

In terms of contextual and sexual risk factors we found that previous migration to the USA was significantly higher among men than women. In terms of sexual identity, 80 % of men reported being homosexual vs. 91 % of women reported being heterosexual.

Table 2 displays crude differences related to self-reported HIV history. We found no significant gender difference in unprotected sex as the presumed mechanism of infection. However, women were significantly more likely to have been infected via blood transfusion or sharing syringes and less likely to have been infected due to condom failure (3.1 % vs. 12.1 %). Significant statistical differences were found in other characteristics within this dimension: women were significantly more likely to have been infected by a stable partner, by a partner with military/police background or imprisonment background, though less likely by a partner with a history of migration to the U.S. Women were more likely to have been tested because they found out that their partner was HIV positive, and only 8 % were diagnosed during pregnancy.

Furthermore, in Table 3 we explore crude differences in terms of sexual behavior and other HIV risk factors before their HIV diagnosis. Men were significantly more involved in transactional sex than women: 19.4 % of men vs. 3.9 % women had paid or received money for sex; and among men, 90.5 % reported that the transaction involved other men. Condom use was less frequent among women. Women were less likely to use alcohol at sexual encounters. Additionally, no differences in percentage of sexual abuse; however women reported more occurrences of it. Mean age at first abuse was younger for men. No difference in the average of sexual encounters in the last month was identified.

**Fig. 1:**
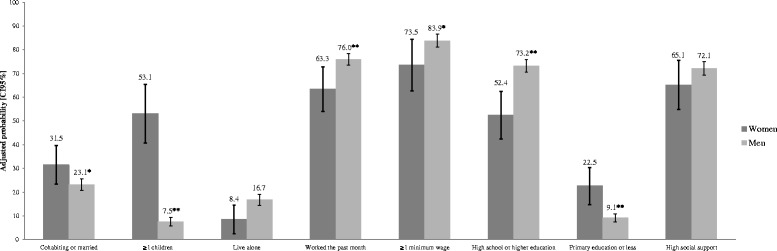
Adjusted probabilities from probit models to selected marginalization, demographic characteristics by sex. Footnote: **p < 0.01 and *p < 0.05. The figure shows estimated adjusted probabilities of occurrence of selected characteristics. All models were controlled for the following socioeconomic and risk characteristics: sex, age, indigenous status, living alone, marital status, number of children, educational level, employment status during the past month, prior incarceration, prior migration to the U.S. Sexual identity was included as a control variable in all the models with socioeconomic variables as dependent variables. When any of these control variables was a dependent variable, it was nor included as control in the model

**Fig. 2:**
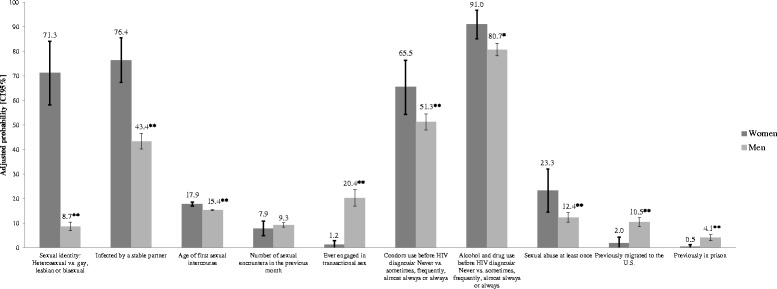
Adjusted probabilities from probit models to selected sexual and other risk characteristics by sex. Footnote: **p < 0.01 and *p < 0.05. The figure shows estimated adjusted probabilities of occurrence of selected characteristics. All models were controlled for the following socioeconomic and risk characteristics: sex, age, indigenous status, living alone, marital status, number of children, educational level, employment status during the past month, prior incarceration, prior migration to the U.S. Sexual identity was included as a control variable in all the models with socioeconomic variables as dependent variables. When any of these control variables was a dependent variable, it was nor included as control in the model

In Figs. 1 and 2 we show the results of the second part of the analysis. We estimated adjusted probabilities of occurrence of selected socioeconomic characteristics, sexual behaviors and other HIV-related risk characteristics, by sex. Our results show that while the HIV epidemic among men is linked mainly to sexual risk behavior, the epidemic among women is mainly linked to social and economic vulnerability and marginalization.

First, we show that adjusting for other demographic, socioeconomic and sexual identity characteristics, HIV-infected women are more likely to be married or living with a stable partner, and more likely to have children. Similarly, HIV-infected women have a lower probability of having been employed in the past month than men; and among those employed, women are less likely to earn more than the minimal wage than men. At the same time, HIV-positive women are significantly more likely to have primary or lower level of education, and less likely to have high school or higher level of education than men.

The picture is completed in Fig. 2, where we present the adjusted probabilities of sexual and other HIV-related risk characteristics. Women are significantly more likely to report being heterosexual than men and also more likely to report being infected by a stable partner. Men report a younger age of first sexual intercourse. The likelihood of having been engaged in transactional sex is significantly higher for men than for women. Women were more likely to never have used condoms before HIV diagnosis and more likely to never have used alcohol at sexual encounters before diagnosis. The adjusted probability of having been sexually abused is significantly higher for women than for men. Men were more likely to have previous experience of both migration to the U.S., and imprisonment than women.

## Discussion

Our results highlight the key structural differences between HIV-positive men and women in Mexico City. Our results are consistent with the hypothesis that the HIV epidemic in Mexico is not one, but two different epidemics: one linked to sexual risk behavior among MSM with relatively high levels of education and low levels of poverty, and a different one among women explained by social vulnerability and economic marginalization. Women in this study are particularly vulnerable in socio-economic terms, while men demonstrate riskier behaviors. Women were more likely to have children, which, in combination with lower levels of formal employment, salary, and lower self-placement on the social ladder points to significant socio-economic vulnerability for both the women and their children.

To our knowledge, this is the first study on gender differences among HIV/AIDS patients in large urban settings in Mexico. Although there is large body of literature addressing these aspects among sex workers, injecting drug users and their partners, and partners of migrants in rural communities, almost no study has been published on women from the “general population” and their risk to get infected with HIV in Mexico. This topic has been well documented in the US with comparable results, more prominently through the body of research recently described as the “vulnerability paradigm” [31] which described the vulnerable position of women towards HIV infection compared with men, as a result of biological differences in susceptibility, sexual autonomy and power dynamics between men and women [33–37].

The study was based in an HIV clinic for the uninsured population, which includes the roughly 50 % of the population with lower socioeconomic status in Mexico. However, while male participants had salaries only marginally less the national average (US$411/month, [38]), women’s average salary was less than half of men’s. The proportion of participants who speak an indigenous language (7.38 %) is five times higher than that of Mexico City’s general population [39], and for women it is almost eight times higher, highlighting the even greater vulnerability of indigenous women who migrated to the national capital.

Women in rural communities have been found to be particularly vulnerable due to unprotected sex with stable partners, citing many reasons for not negotiating condom use [14]. Women in Mexico City may face the same problem – female participants were significantly less likely to use condoms and had a greater (self-reported) probability of acquiring HIV from a stable partner, which may reflect their difficulty negotiating condom use in stable relationships.

Female participant’s vulnerability is further demonstrated by the greater amount of sexual abuse they have suffered. IPV is a known factor in women’s vulnerability to HIV [8, 16] and though not all forms of IPV were assessed, women reported that the perpetrator of sexual abuse was more likely to have been a partner, or someone well-known to them. Furthermore, they were more vulnerable to infection by military/police men, or former prisoners. Previous studies have found high levels of unprotected sex with other men among the male partners of HIV positive women [9]. The risk behaviors of male partners of women in the study were not assessed, but 7 % of male participants reported having sex with men and women. A previous study of MSM in Mexico found that 17.6 % self-identified as bisexual and 41 % reported previous sex with women [7, 10].

MSM are known to be at greater risk of HIV for several reasons [9]. Almost 90 % of men in this study were MSM, and despite more consistent condom use than women, 50 % reported never using condoms before diagnosis, a substantial risk for MSM, the group among which HIV prevalence is highest in Mexico. Men demonstrated higher risk behavior in reporting greater involvement in transactional sex. Migration, another known factor in increasing risk behavior [11], was more commonly reported by men than women – both in terms of personal history and of the person who they reported to have infected them.

The study was limited by its cross-sectional design, allowing for only statistical associations rather than causal inferences to be made, and by the fact that all behaviors were self-reported. Our results may thus be subject to bias and measurement error, particularly in relation to the source of infection. A larger percentage of women were excluded from the study due to missing data. The reason for this is unknown, but anecdotal information from the field during data collection suggests that it may reflect increased time pressure for women and the feeling of decreased ability to complete the questionnaire due to higher illiteracy, impacting women’s ability to complete the survey, which may have excluded some of the most vulnerable women, thus potentially biasing downwards the difference between men and women found in the analysis. This implies that our results are likely a conservative estimation of the social vulnerability of women in this study.

Though the information was treated as confidential, characteristics such as drug use and sex work may have been under-reported due to fear of legal implications. Information on experience of sexual violence may also be under-reported due to its distressing nature. However, we do not expect that these possible biases would be systematically different between men and women.

Despite these limitations, this study has shed light on differences by gender in the HIV epidemic in Mexico City and suggests that there are two epidemics in Mexico, pointing to the vulnerability of women and the risky sexual behavior of men. Identifying factors contributing to both vulnerability and risk will help guide both prevention and treatment efforts. MSM are particularly at risk, and prevention strategies should continue to target this group, focusing on risk reduction, particularly greater use of condoms and HIV testing for earlier diagnosis and linkage to care and treatment. Sex workers and migrants appear to be particularly important subpopulations in prevention strategies.

The strategies to address the epidemic among women need to be different. Universal HIV testing for pregnant women and easy access to testing for all other women should probably be a central component of the strategy. Additionally, finding effective ways to increase awareness among women of the risk of HIV and higher access to preventive methods such as condoms and HIV testing for those without a stable partner, should also be a priority. Perhaps more importantly, there is an urgent need to encourage men living with HIV to disclose to their partners, and to encourage couples testing, however there is no evidence on how to effectively do it. Researchers and policy makers need to find ways to do this respecting human rights of people living with HIV but also placing women’s health as a high priority.

Additionally, it is important to change the perception of the healthcare community in Mexico of virtually no risk for HIV infection among women. There must be concerted efforts to encourage testing for victims of sexual abuse. A higher attention should be given to women in higher risk groups such as those with partners having sex with men and women and partners with a military, police or prison background. Early counseling to encourage disclosure by men living with HIV with female partners should be encouraged [40]. Long-term follow-up should be arranged for sero-discordant couples to provide access to regular screening, counseling and condoms and for sero–concordant couples for monitoring of their sexual and reproductive health needs.

Those providing treatment also need to recognize the socio-economic vulnerability of women and their children. Poverty plays an important role for both sexes but women in the study were significantly more affected.

## Conclusion

Significant differences exist between men and women with HIV in Mexico City. Women live in a context of high social and economic vulnerability; while men are characterized by riskier sexual behavior. Therefore, the strategies to address the epidemic among women need to be different than those typically implemented in Mexico and other countries with concentrated epidemics. More complex interventions targeting other determinants beyond sexual behavior are needed. Strategies should pay particular attention to the most vulnerable women and include the provision of information and services in indigenous languages if needed. Furthermore, our results suggest that other structural components must be considered as well and that prevention of HIV is yet another reason to improve women’s access to education on reproductive rights, on sexual and reproductive health, and to increase access to economic opportunities and improved living conditions.

However, because both sexes face significant socio-economic disadvantage and stigma, gender issues must be addressed in both prevention and treatment efforts, focusing on equality at work and home, and ensuring access to social protection services. Future strategies to increase awareness, diagnosis and treatment should take this into account in order to try and maximize impact, and monitoring and evaluation should be carried out with attention to gender specific differences.
